# The seizure‐inducing plastic explosive RDX inhibits the *α*1*β*2*γ*2 GABA_A_ receptor

**DOI:** 10.1002/acn3.51536

**Published:** 2022-03-24

**Authors:** Brandon Pressly, Ruth D. Lee, Vikrant Singh, Isaac N. Pessah, Heike Wulff

**Affiliations:** ^1^ Department of Pharmacology School of Medicine University of California Davis California USA; ^2^ Department of Molecular Biosciences, School of Veterinary Medicine University of California Davis California USA

## Abstract

**Objective:**

Royal demolition explosive (RDX) can induce seizures in wildlife and humans following release into the environment or after voluntary consumption. During the Vietnam War, RDX intoxication was the most common cause of generalized seizures in US service personnel, and in some sections of the armed forces, eating of RDX has continued as “a dare” to this day. After its mechanism of action was long unknown, RDX was recently shown to be a GABA_A_ receptor antagonist. We here determined the GABA_A_ receptor subtype‐selectivity of RDX and mapped its functional binding site.

**Methods:**

We used whole‐cell patch‐clamp to determine the potency of RDX on 10 recombinantly expressed GABA_A_ receptors and mapped the RDX binding site using a combination of Rosetta molecular modeling and site‐directed mutagenesis.

**Results:**

RDX was found to reversibly inhibit the *α*1*β*2*γ*2 GABA_A_ receptor with an IC_50_ of 23 *μ*mol/L (95% CI 15.1–33.3 *μ*mol/L), whereas *α*4 and *α*6 containing GABA_A_ receptor combinations were 4–10‐fold less sensitive. RDX is binding to the noncompetitive antagonist (NCA) site in the pore. In a molecular model based on the cryo‐EM structure of the resting state of the *α*1*β*2*γ*2 receptor, RDX forms two hydrogen bonds with the threonines at the T6’ ring and makes hydrophobic interactions with the valine and alanine in 2′ position of the *α*1 or *β*2 subunits.

**Interpretation:**

Our findings characterize the mechanism of action of RDX at the atomistic level and suggest that RDX‐induced seizures should be susceptible to treatment with GABA_A_ modulating drugs such as benzodiazepines, barbiturates, propofol, or neurosteroids.

## Introduction

The high‐energy explosive hexahydro‐1,3,5‐trinitro‐1,3,5‐triazine, also known as cyclonite or RDX (Royal Demolition Explosive), is the major component of plastic explosives such as composition C‐4 which contains ~90% RDX and ~10% plasticizers.^1^ Without a detonator, RDX is relatively insensitive to impact and can be transported and even burned without exploding. Due to these properties, RDX has been extensively employed in every major war since World War II and is also widely used for civilian purposes. In 2018 RDX had an estimated market of $10.4 billion.^2^ RDX release as waste during manufacture, via explosion of ordinances, and during disposal of munitions constitutes an environmental concern for exposure of both humans and wildlife.^2^, ^3^ At 32 Environmental Protection Agency (EPA) listed National Priority Sites, RDX has been found to leach into soil and groundwater and, in some cases, to induce seizures in fish, rodents, lizards, or birds.^3^, ^4^, ^5^ There also is suggestive evidence of carcinogenic potential based on an EPA assessment performed in 2018.^3^

In addition to these chronic environmental exposures, acute RDX intoxications have occurred in workers in ammunition factories, in soldiers who used RDX as cooking fuel or voluntarily consumed it.^1^, ^6^ Following oral or inhalation exposure, RDX causes dizziness, headaches, vomiting, and confusion and, at high doses, generalized tonic–clonic seizures. During the Vietnam War, it apparently was common knowledge among field troops that small quantities of RDX would produce a “high” similar to ethanol.^1^ When gram quantities were consumed with standard issued beer, soldiers supposedly could achieve increased inebriation, but often went on to develop seizures that required hospitalization. In a report from 1969, army physicians recognized RDX intoxication as the most common cause of generalized seizures in US service personnel in Vietnam.^1^ “Eating” of RDX has continued in some sections of the armed services to this day as “a dare” or rite of passage.^7^ For example, in 2019, a 22‐year‐old active‐duty male who was training with explosives was documented to exhibit status epileptics with RDX plasma concentrations of 3.06 *μ*g/mL following consumption of a piece of C‐4.^8^ Another case of a 20‐year old combat engineer on a demolition range eating C‐4 under peer pressure was reported as we were writing this paper.^9^

The molecular mechanism of RDX action was long unknown, and its elucidation was initially confused by anecdotal reports that RDX increased salivation and lacrimation^10^ similar to organophosphate pesticides or nerve agents targeting acetylcholine esterase (AChE). In a seminal study in 2011, Williams et al. clarified this question by demonstrating that RDX administration to rats does not result in salivation or inhibition of brain AChE.^11^ Instead, RDX was shown to displace [^35^S]‐*t*‐butylbicyclophosphoorothothionate (TBPS) in rat brain membrane preparations with a *K*_i_ of 21 *μ*mol/L and to inhibit GABA‐induced currents in basolateral amygdala neurons. This suggested that RDX is binding to the noncompetitive antagonist (NCA) site in the pore of GABA_A_ receptors similar to picrotoxinin.^11^ Using molecular modeling and site‐directed mutagenesis, we here confirm that RDX is binding to the NCA site in the pore of GABA_A_ receptors, specifically to the so‐called threonine ring where all five subunits of the GABA_A_ receptor channel contain a pore lining threonine in 6′ position in the M2 segment. In whole‐cell patch‐clamp experiments with 10 recombinantly expressed GABA_A_ receptors, we also determined the subtype‐selectivity of RDX and found that it blocks *α*1*β*2*γ*2, the most common GABA_A_ receptor combination in the mammalian CNS, with an IC_50_ of 23 *μ*mol/L in a fully reversible manner. Our findings thus characterize the mechanism of action of RDX at the molecular level and suggest that RDX‐induced seizures should be susceptible to treatment with GABA_A_ modulating drugs such as benzodiazepines, neurosteroids, propofol, or barbiturates.

## Materials and Methods

### Chemicals

RDX (Hexahydro‐1,3,5‐trinitro‐1,3,5‐triazine) was purchased as certified reference material (Cerilliant® from MilliporeSigma) in a 1‐mg/mL solution in acetonitrile (1 mL ampules, chromatographic purity 99.9%). For electrophysiological experiments, the acetonitrile was evaporated and RDX immediately reconstituted in 1 mL DMSO rendering 0.1 molar stocks. RDX waste was treated with nitric acid and disposed of using the waste accumulation program at UC Davis. Bicuculline, picrotoxinin, and GABA were purchased from MilliporeSigma. Diazepam was purchased from Tocris Bioscience.

### Clones and cell lines

The sources and recombinant expression of the human GABA_A_ receptors *α*1, *α*2, *α*4, *α*6, *β*1, *β*3, *γ*1, *γ*2L, and *δ* and the rat GABA_A_ receptor *β*2 were previously described.^12^ Briefly, GABA_A_ receptors were expressed in L929 cells, a mouse fibroblast cell line (CCL‐1, American Type Culture Collection, Manassas, VA). Cells were transfected using FuGENE 6 (ThermoFisher) transfection reagent with an equal amount of each of the subunits (1:1:1) in combination with green fluorescent protein (GFP) expressed from the pEGFP‐C1 vector (Invitrogen). Cells were detached by trypsinization 48 h post‐transfection, washed, and plated onto poly‐L‐lysine coated glass coverslips. Transfected cells were identified as GFP‐expressing cells, using an epifluorescence microscope for the whole‐cell voltage‐clamp studies. Correct subunit composition of the various GABA_A_ receptors was tested by their sensitivity to diazepam, propofol, allopregnanolone, DS2, fipronil, bicuculline, and Zn^2+^ as previously described.^12^

### Electrophysiology

Whole‐cell voltage‐clamp experiments were performed with an EPC‐10 HEKA amplifier (HEKA Elektronik) as previously described.^12^ Cells were bathed in an external Ringer solution consisting of 160 mmol/L NaCl, 4.5 mmol/L KCl, 1 mmol/L MgCl_2_, 2 mmol/L CaCl_2_, 10 mmol/L HEPES, pH 7.4, 310 mOsm. Recording electrodes were pulled from soda lime glass micro‐hematocrit tubes (Kimble Chase, Rochester, NY) and fire‐polished to resistances of 1.6–2.9 MΩ. Electrodes were filled with an internal solution consisting of 154 mmol/L KCl, 2 mmol/L CaCl_2_, 1 mmol/L MgCl_2_, 10 mmol/L HEPES and 10 mmol/L EGTA, pH 7.2, and 301 mOsm. Cells were voltage‐clamped at −80 mV, and currents recorded under the local 5‐sec applications of varying GABA concentrations to the patch‐clamped cell using an 8‐channel pinch valve controlled gravity perfusion system (VC3‐8xG system, ALA Scientific). The GABA concentration–response relationships for the GABA_A_ receptor subunit combinations used here were previously published by our group,^12^ and the blocking potency of RDX was tested at the respective GABA EC_90_. RDX additions and washes were performed through a separate, syringe‐driven perfusion system and with a volume (2 mL) that exchanged the chamber volume five times. Varying concentrations of RDX were allowed to sit for 3 min on the cell before re‐application of EC_90_ GABA directly onto the cell through the gravity perfusion system. For testing or washing out of RDX concentrations of 100 *μ*mol/L or more we added 1% of DMSO to the Ringer solution to keep RDX in solution. One cell was used per concentration. Cells that became leaky during the experiment or that did not produce the same magnitude of response to EC_90_ GABA twice before the experiment and after washout of RDX were excluded from the analysis. For analysis of current blockade, the area under the current curve (AUC_Max_) was determined for the control (EC_90_ GABA) and the AUC_Ex_ after exposure. [AUC_Ex_]/[AUC_Max_] × 100 = % Current Blocked. Data analysis and data fitting to the Hill equation to obtain IC_50_ values was performed using GraphPad Prism8 (GraphPad Software, La Jolla, CA). Individual data points are presented as mean ± SD from 5 to 8 independent recordings. IC_50_ values are presented with 95% confidence intervals. For screening of mutant channels 100 *μ*mol/L RDX and EC_90_ GABA were used to evaluate if the mutation affected RDX potency. Percentage of current blocked (mean ± SD from *n* = 5–8 cells per mutant) was analyzed with one‐way ANOVA followed by Dunnett's test to compare the means to the WT control and to correct for multiple comparisons. *p < 0.05, **p < 0.01, ***p < 0.001.

### Molecular modeling

Using the Rosetta molecular modeling suite^13^ with membrane environment‐specific energy functions^14^, ^15^ we generated a model of the *α*1*β*2*γ*2 GABA_A_ receptor in the resting state based on the cryo‐EM structure of the *α*1*β*3*γ*2 receptor^16^ with picrotoxinin bound (pdb id: 6X40). Structural refinement and docking of RDX with the RosettaLigand application^17^ using the Talaris2014 energy function was performed as previously described in detail.^18^ Molecular graphics were rendered with the UCSF Chimera software (Resource for Biocomputing, Visualization, and Informatics, San Francisco, CA) (Pettersen et al., 2004). Protein Data Bank (pdb) format files of the model are available upon request.

### Mutagenesis

Mutagenesis primers (20–30 base pairs in length, with a 5–8 base pair overhang on the 3′ end) were designed with PrimerX software (http://www.bioinformatics.org/primerx), synthesized by ThermoFisher, and mutagenesis performed as previously described.^18^ Mutant sequences were confirmed via sequencing using ABI 3730 Capillary Electrophoresis Genetic Analyzers (UC Davis DNA Sequencing Facility). Mutant *α*1*β*2*γ*2 GABA_A_ receptors were deemed functional if they produced at least 200 pA of current in response to 100 *μ*mol/L GABA and were sensitive to positive modulation by diazepam. The following mutants did not produce functional currents in our hands: *α*1L9’F, *α*1L9’C, *β*2A2’E, *β*2L9’Q, *β*2L9’F, *β*2L9’G, and *β*2L9’V.

### Plasma protein binding

RDX plasma protein binding was determined with rat plasma in triplicate using rapid equilibrium devices with a molecular weight cutoff of 8 kD (RED, Fisher Scientific). RDX concentrations were determined by liquid chromatography using Hewlett Packard 1100 series HPLC equipped with a C‐18 Zorbax Eclipse XDB column (5 *μ*m, 4.6 × 150 mm, Agilent) and an isocratic mobile phase consisting of 50/50 water/acetonitrile for 6.0 min at 25°C. RDX had a retention time of 3.1 min, and UV absorption was monitored 234 nm.

## Results

### RDX displays selectivity for *α*1*β*2*γ*2 among recombinantly expressed GABA_A_ receptors

**Figure 1:**
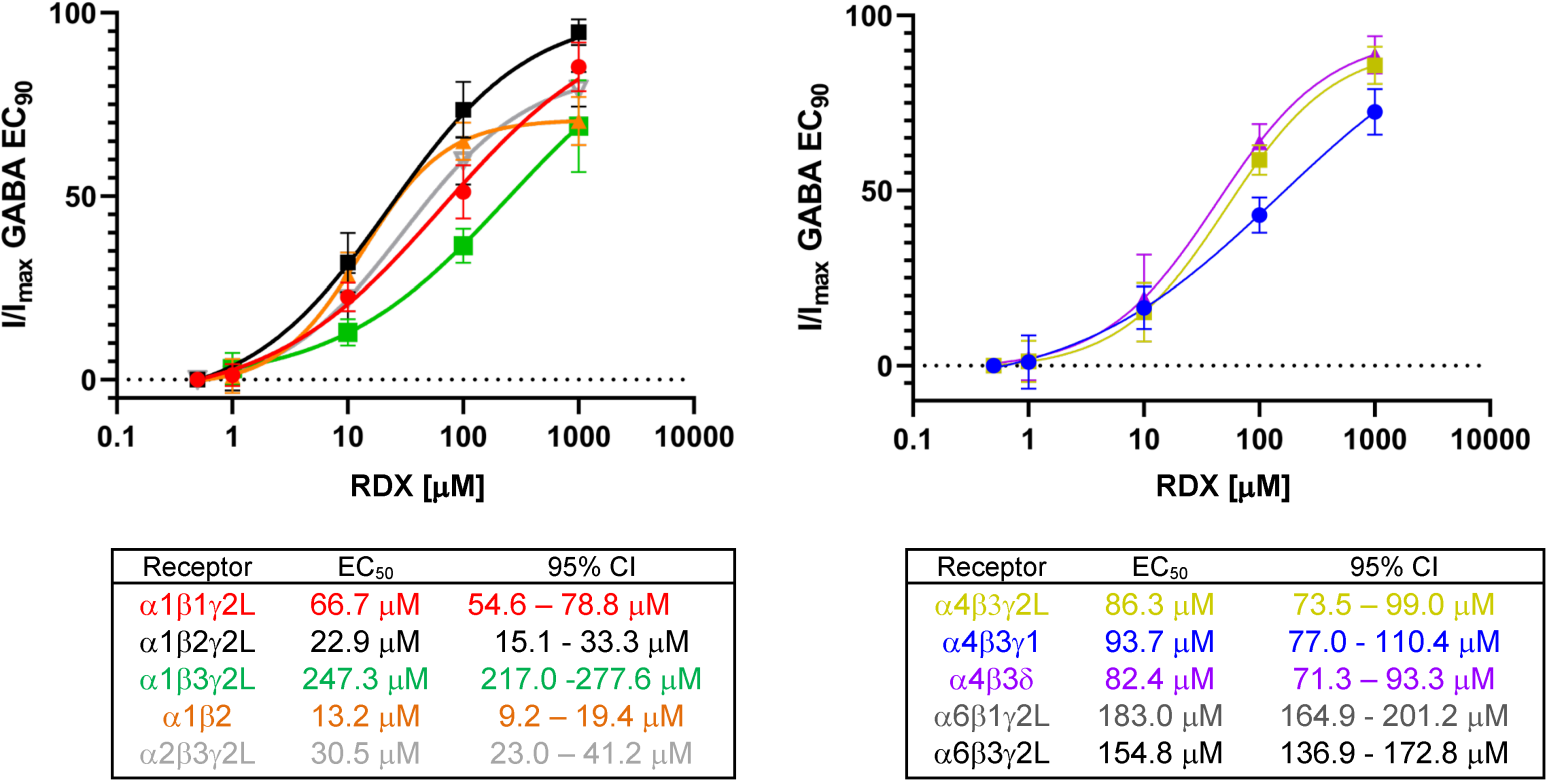
Concentration–response curves and IC_50_ values for RDX‐mediated inhibition of currents evoked by EC_90_ GABA for *α*1 and *α*2 (left) or *α*4 and *α*6 (right) containing GABA_A_ receptors. Data points are mean ± SD from 3 to 8 independent recordings. IC_50_ values are presented with 95% confidence intervals. The EC_90_ GABA concentration was 110 *μ*mol/L for *α*1*β*1*γ*2L, 100 *μ*mol/L for *α*1*β*2*γ*2L, 60 *μ*mol/L for *α*1*β*3*γ*2L, 40 *μ*mol/L for *α*1*β*2, 50 *μ*mol/L for *α*2*β*3*γ*2L, 10 *μ*mol/L for *α*4*β*3*γ*2L, 10 *μ*mol/L for *α*4*β*3*γ*1, 5 *μ*mol/L for *α*4*β*3*δ*, 15 *μ*mol/L for *α*6*β*1*γ*2L, and 10 *μ*mol/L for *α*6*β*3*γ*2L. [Colour figure can be viewed at wileyonlinelibrary.com]

We previously recombinantly expressed and characterized a panel of synaptic and extrasynaptic GABA_A_ receptor subtype combinations.^12^ Correct incorporation of *γ* and *δ* subunits was confirmed by their respective sensitivity to diazepam, propofol, allopregnanolone, DS2, fipronil, bicuculline, and Zn^2+^.^12^ The GABA concentration–response curves were in good agreement with literature.^12^ We here used these established assays to study RDX. For each subtype combination, increasing concentrations of RDX were tested for their ability to inhibit chloride currents elicited by the GABA EC_90_ for this respective receptor subtype in whole‐cell patch‐clamp experiments. RDX was found to be most potent on the *α*1*β*2*γ*2 subtype combination (Fig. 1) which was blocked with an IC_50_ of 22.9 *μ*mol/L (95% CI 15.1–33.3 *μ*mol/L, Emax 95%). RDX blocked GABA_A_ receptors consisting of only *α*1*β*2 with slightly higher potency (IC_50_ 13.2 *μ*mol/L) but reduced efficacy (Emax ~70%) and was 3–10‐fold less potent on *α*1 receptor combinations containing *β*1 or *β*3 subunits (Fig. 1). GABA_A_ receptors containing *α*4 and *α*6 subunits (either with *γ* or *δ* subunits) were 4–8‐fold less sensitive to RDX than *α*1*β*2*γ*2 receptors (Fig. 1), while there was no significant selectivity over *α*2*β*3*γ*2 receptors.

### RDX is a fully reversible GABA_A_ receptor blocker

**Figure 2:**
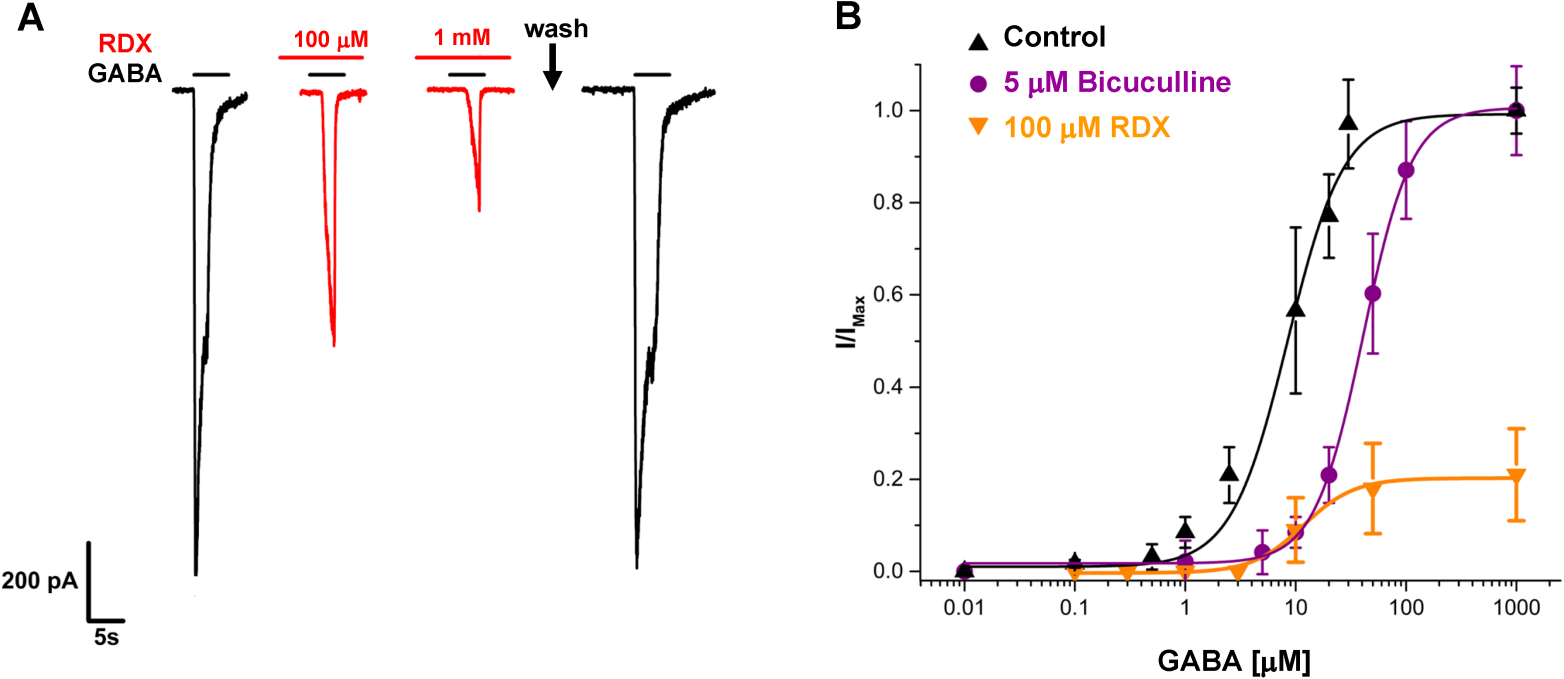
RDX is a reversible, noncompetitive inhibitor of the *α*1*β*2*γ*2L GABA_A_ receptor. (A) Example recording showing that RDX is reversible on washout. Currents were elicited by 100 *μ*mol/L GABA. (B) Comparison of the effects of bicuculline (5 *μ*mol/L) and RDX (100 *μ*mol/L) on the GABA concentration–response curve. Data points are mean ± SD from 3 to 8 independent recordings. [Colour figure can be viewed at wileyonlinelibrary.com]

We next probed the mechanism of RDX inhibition in more detail. When determining the subtype‐selectivity of RDX, we had observed that RDX was more potent when GABA_A_ receptors were preincubated with RDX before GABA application than if GABA and RDX were applied simultaneously. As shown in Figure 2A, if *α*1*β*2*γ*2L receptors were first activated by application of 100 *μ*mol/L GABA directly to the patch‐clamped cell to elicit a control current and RDX was then perfused into the recording chamber following washout of GABA, reapplication of GABA after 3 min of incubation with RDX induced a smaller current with virtually no further enhancement of current decay during the 5‐sec duration of the GABA application. RDX inhibition was fully reversible by a washout with 2 mL of external Ringer solution. With these fast kinetics, especially the quick washout, RDX resembles the pore blockers picrotoxinin^16^, ^19^ and TETS^18^ suggesting that it could be binding to the NCA site in keeping with its ability to displace [^35^S]‐TBPS in binding assays with rat brain membrane preparations.^11^ In order to confirm that RDX is indeed a noncompetitive antagonist we next compared the effect of RDX and the competitive antagonist bicuculline on the GABA concentration–response curve for the *α*1*β*2*γ*2L receptor (Fig. 2B). While bicuculline shifted the curve to the right without decreasing the maximum GABA response in keeping with its binding to the same site as GABA, 100 *μ*mol/L of RDX drastically reduced the maximum response elicited by 50 *μ*mol/L or even 1 mM GABA demonstrating that RDX indeed behaves like a NCA that cannot be competed off by GABA.

### RDX positively enhances GABA activity at low concentrations

**Figure 3:**
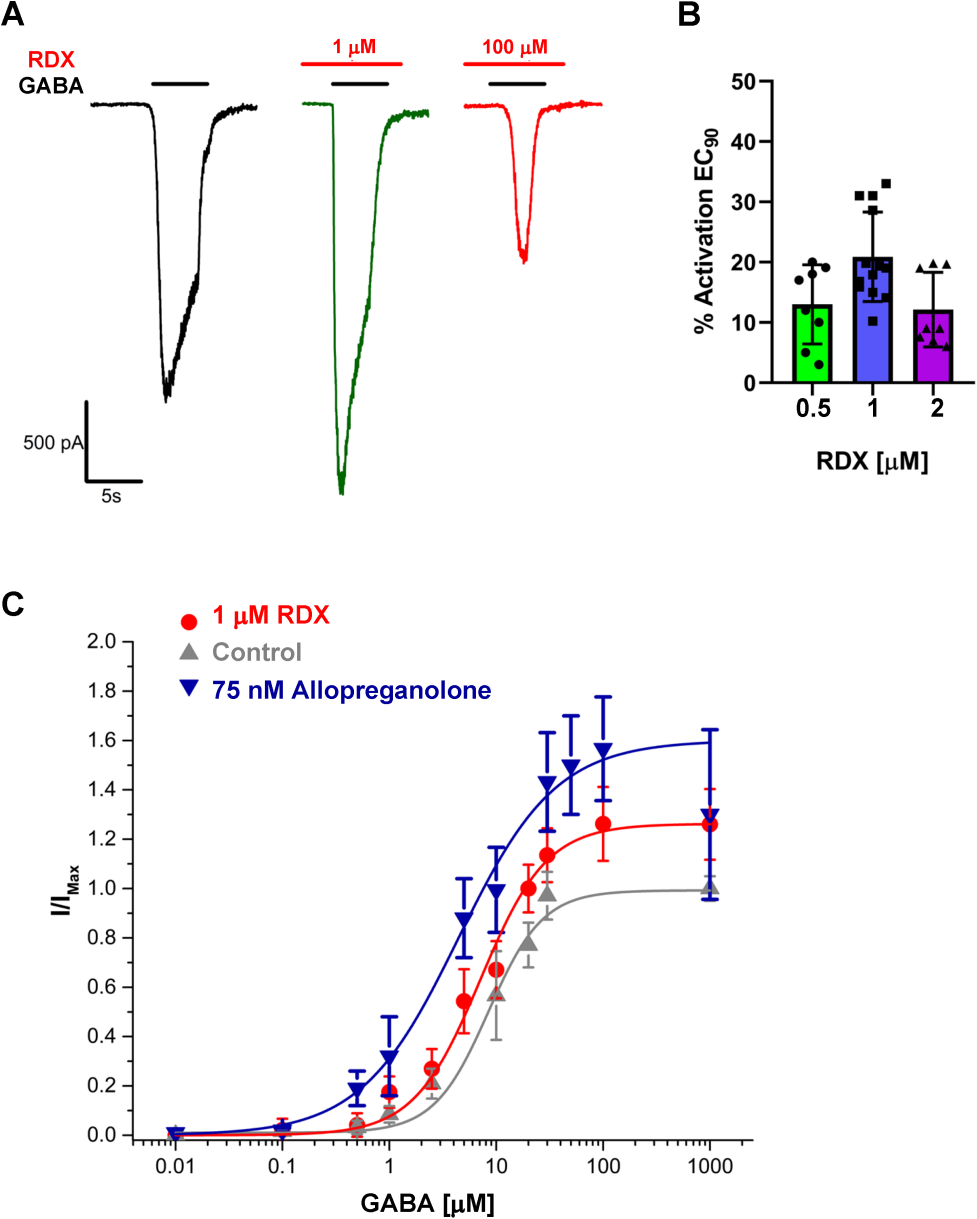
RDX enhances GABA effects at low concentrations. (A) Example recording showing that 1 *μ*mol/L of RDX enhances chloride currents elicited by 100 *μ*mol/L GABA while 100 *μ*mol/L RDX inhibits currents. (B) Scatterplots showing the percentage of current activation by 0.5, 1, and 2 *μ*mol/L of RDX (n = 8 to 13 cells, error bars are ±SD). (C) Comparison of the effects of allopregnanolone (75 nmol/L) and RDX (1 *μ*mol/L) on the GABA concentration–response curve of the *α*1*β*2*γ*2L GABA_A_ receptor. Data points are mean ± SD from 3 to 8 independent recordings. [Colour figure can be viewed at wileyonlinelibrary.com]

Interestingly, in addition to blocking GABA‐induced currents through the *α*1*β*2*γ*2 receptor at high *μ*mol/L concentrations, RDX potentiates currents induced by GABA EC_90_ at low concentrations (Fig. 3A). This GABA potentiating effect occurred in a concentration range of between 500 nmol/L and 2 *μ*mol/L as shown in Figure 3B and in Figure S1, which contrasts the biphasic effects of RDX with the straightforward inhibitory effects of picrotoxinin. A full GABA concentration–response curve, obtained in the presence of 1 *μ*mol/L RDX, revealed that the potentiating effects of RDX were most pronounced at saturating GABA concentrations (Fig. 3C). Low‐concentration RDX thus differs in its behavior from benzodiazepines and somewhat resembles the neurosteroid allopregnanolone. However, unlike allopregnanolone, which changes from being a positive allosteric modulator (PAM) to being a direct activator at higher concentrations, higher concentrations of RDX have no effect in the absence of GABA and block GABA‐induced currents in the presence of GABA (Figs. 1 and 2). A similar GABA potentiating effect at low RDX concentrations was observed on all other tested GABA_A_ receptor combinations including the *α*4*β*3*δ* receptor (Fig. S1).

### Docking of RDX into a model of the *α*1*β*2*γ*2 GABA_A_ receptor with the RosettaLigand method

**Figure 4:**
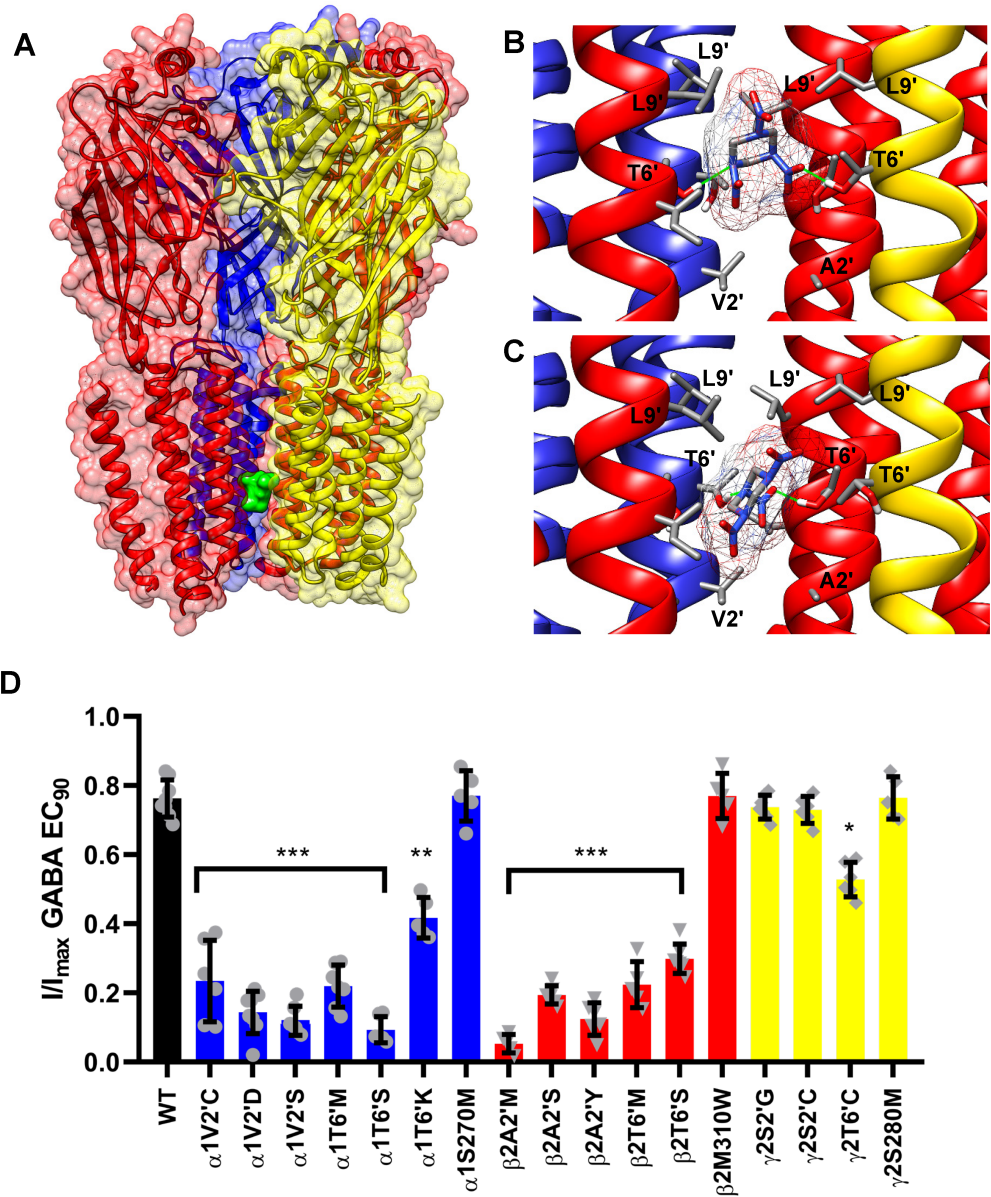
The RDX binding site. (A) Molecular model of the RDX binding site in the pore of the *α*1*β*2*γ*2 receptor identified of RosettaLigand. The receptor is color‐coded as follows: *α*1 (blue), *β*2 (red), and *γ*2 (yellow). RDX is shown as a green space‐filled model. One *α*1 subunit is removed to allow a view of the permeation pathway. (B and C) Closeup views of the two alternative, low‐energy binding poses of RDX at T6’ ring. RDX is shown in stick representation, hydrogen bonds are shown in green. (D) Site‐directed mutagenesis of the *α*1*β*2*γ*2L receptor. Percentage of current blocked by 100 *μ*mol/L RDX (mean ± SD from *n* = 5–8 cells per mutant) was analyzed with one‐way ANOVA followed by Dunnett's test to compare the means to the WT control and to correct for multiple comparisons. *p < 0.05, **p < 0.01, ***p < 0.001. [Colour figure can be viewed at wileyonlinelibrary.com]

We next decided to probe the mechanism of RDX action at the atomistic level by docking RDX into a model of the resting state of the *α*1*β*2*γ*2 GABA_A_ receptor with the RosettaLigand application. Our model was based on the recently published cryo‐electron microscopy structure of the human *α*1*β*2*γ*2 receptor in lipid nanodiscs.^16^ The receptor had been captured in multiple states including a probably resting state structure with picrotoxinin in the pore at a resolution of 2.86 Å (pdb id: 6X40). We removed picrotoxinin, refined the structure, allowed the side chains to relax, and then docked RDX in 50,000 random starting positions in the pore. Rosetta identified a binding site in the permeation pathway (Fig. 4A) with two alternative, frequently sampled, and low‐energy binding poses (Fig. 4B and C). In both poses, RDX is accepting two hydrogen bonds from the T6’ ring (residues are counted starting from the cytoplasmic entry to the pore) where all five subunits have a threonine at the same position in their highly homologous M2 segments. In the first binding pose, RDX is positioned somewhat “higher up” and is making hydrophobic van der Waals interactions with the leucines at the L9’ ring and accepting two hydrogen bonds at the T6’ ring from the *α*1 and *γ*2 subunits, which are colored blue and yellow, respectively, in Figure 4B. In the second binding pose, RDX is hydrogen bonding with two other threonine residues at the T6’ ring, *β*2T6’, and *α*1T6’, while one nitro‐group is turned “down” toward the 2′ ring where it is interacting with *α*1V2′ and *β*2A2’.

### RDX exerts its inhibitory activity by binding to the T6′ ring of the *α*1*β*3*γ*2 GABA_A_ receptor

**Figure 5:**
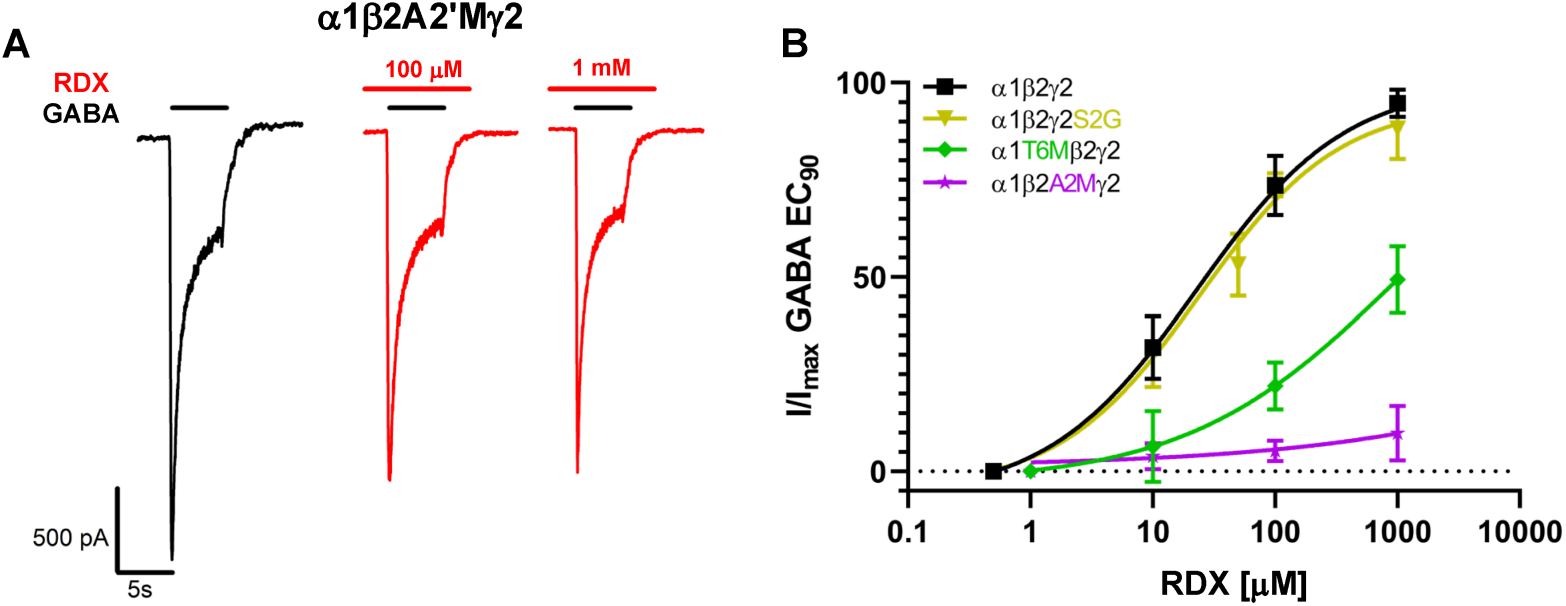
Representative examples from the mutagenesis. (A) The *β*2A2’M mutant is insensitive to RDX. Currents were elicited by 100 *μ*mol/L GABA. (B) RDX concentration–response curves for the wild‐type *α*1*β*2*γ*2L receptor and the *α*1T6’M, *β*2A2’M, and *γ*2S2’G mutants. Data points are mean ± SD from 3 to 8 independent recordings. [Colour figure can be viewed at wileyonlinelibrary.com]

In order to determine whether RDX is interacting with any of the residues identified by RosettaLigand we mutated the respective residues as well as control residues in the *α*1*β*2*γ*2L GABA_A_ receptor and compared the ability of RDX at 100 *μ*mol/L, which corresponds to IC_80_, to block chloride currents carried by wild‐type and mutant channels (Fig. 4D). When interpreting the results, it should be kept in mind that mutations in the *α* or *β* subunit always introduce changes in two of the five subunits of the heteropentameric channel consisting of two *α*, two *β*, and one *γ* subunit. We studied a total of 17 mutations. For the *α*1 subunit we made seven mutation: six mutations in the NCA site (V2’C, V2’D, V2’S, T6’M, T6’K, and T6’S) and one mutation (S270M) outside of the pore. For the *β*2 subunit, we studied a total of six mutants: T6’S, A2’M, A2’Y, A2’S, T6’M, and M310W. All *α*1 or *β*2 mutations at the 2′ and 6′ ring significantly reduced the response to 100 *μ*mol/L RDX (Fig. 4D). For the *γ*2 subunit, which only contributes one subunit to the pentameric receptor, we made four mutations: S2’C, S2’G, and T6’C and S280M. While mutating S2′ had no effect, the *γ*2T6’C mutant reduced RDX potency, but not as drastically as the *α*1 or *β*2 T6′ mutations in keeping with the fact that *γ*2 only contributes one residue at the 2′ and 6’ ring. Figure 5 shows representative currents and concentration–response curves from the *α*1T6’M, *β*2A2’M, and *γ*2S2’G mutants. Our modeling had also shown possible interactions for RDX at the L9′ ring, but unfortunately all the L9′ mutations we were able to generate failed to express functional channels. Control mutations at the top of the TMD domain in the M3‐M1 interfaces in the phenobarbital binding site^16^ did not affect RDX potency (Fig. 4D). Taken together, our mutagenesis studies demonstrate that RDX is binding to the NCA site in the pore of the *α*1*β*2*γ*2 GABA_A_ receptor and confirm the RosettaLigand predictions of molecular interactions at the 2′ and 6′ ring.

While we were thus able to identify the site of action for the inhibitory activity of RDX, our attempts of identifying the site mediating its GABA potentiating activity at low concentrations failed. None of the mutations reducing the inhibitory effect of RDX affected its potentiator activity at low concentrations (data not shown) indicating that the potentiator effect is mediated through a different site. However, none of the mutations that are known to render propofol or barbiturates (*α*S270M, *β*2M310W, and *γ*2LS280W) less active or inactive,^20^ reduced the activating activity of RDX in our hands suggesting that RDX is exerting its GABA potentiating activity through a novel site, which we were not able to identify through molecular modeling or mutagenesis.

## Discussion

RDX, the major component of plastic explosives, induces seizures following accidental or intentional consumption. RDX intoxication therefore should be on the differential diagnosis for new onset seizures in military personnel or anybody handling explosives.^8^ Using a combination of electrophysiology, molecular modeling, and site‐directed mutagenesis, we here show that RDX inhibits the *α*1*β*2*γ*2 GABA_A_ receptor with an IC_50_ of 23 *μ*mol/L by binding to the threonine ring in the channel pore, at the same site as picrotoxinin.^16^, ^21^ In comparison to picrotoxinin, which we previously tested by electrophysiology on the same recombinantly expressed GABA_A_ receptor subtype combinations,^12^ RDX is roughly eightfold less potent but somewhat more selective in its preference for the *α*1*β*2*γ*2 subtype. Since *α*1‐subunit containing GABA_A_ receptors are most highly expressed in the cortex, thalamus, pallidum, and hippocampus, and the *α*1*β*2*γ*2 subunit combination is estimated to constitute about 60% of all GABA_A_ receptors,^22^, ^23^ this subtype combination likely constitutes the most relevant pharmacological target for the seizure‐inducing activity of RDX. The second most abundantly expressed GABA_A_ subtype combination, *α*2*β*3*γ*2, is only slightly less sensitive to RDX (IC_50_ 30 *μ*mol/L) and therefore is also likely to contribute to its pharmacodynamic activity following intoxication.

In a pharmacokinetic/pharmacodynamic correlation study in rats, Burdette et al. demonstrated in 1988 that oral RDX administration at doses of 25 and 50 mg/kg resulting in plasma concentrations of 5–8 *μ*g/mL (22–36 *μ*mol/L) induced intense and sustained seizure activity. In humans, an explosive ordinance who had consumed 1.6 g of RDX (~20 mg/kg) and who exhibited persistent seizure activity for 2 days, has been documented to have RDX plasma concentrations of 3.06 *μ*g/mL (13.77 *μ*mol/L) while showing frontal intermittent rhythmic delta activity with intermittent sharp waves on the EEG.^8^ Another study in rats measured RDX blood and cortical tissue concentrations and found that RDX penetrates well into the brain and achieves brain concentrations that are roughly twofold higher than plasma concentrations.^11^ A brain concentration of 8 *μ*g/g (36 *μ*mol/L) was more than sufficient to initiate seizures.^11^ In order to correlate these brain and plasma concentrations to the functional activity observed in our patch‐clamp assays, we determined the plasma protein binding of RDX using equilibrium dialysis and found that when using rat plasma RDX is 40.0 ± 2.7% protein bound leaving 60% free. Considering this low protein binding, the good brain penetration, and the observation, that for other GABA_A_ antagonists like picrotoxinin, brain concentrations that are able to block roughly 10–20% of GABA_A_ currents in electrophysiological experiments are sufficient to trigger seizures,^24^ the brain concentrations (16–27 *μ*mol/L) that are likely to have been achieved in the above described patient^8^ with matched EEG and RDX plasma levels would have been sufficient to block 10–50% of *α*1*β*2*γ*2‐mediated GABA_A_ currents. The brain concentrations found in rats exhibiting seizures, which ranged from 36 to 45 *μ*mol/L,^11^ are well above the IC_50_ for *α*1*β*2*γ*2 receptors and are also sufficient for significantly inhibiting *α*2*β*3*γ*2 receptors.

RDX had been previously suggested to bind to the NCA site in the pore of GABA_A_ receptors based on the observation that it can displace [^35^S]‐TBPS in binding assays with rat brain membranes.^11^ We here confirmed this hypothesis by taking advantage of the tremendous advances that have been made in the last few years in elucidating GABA_A_ receptor channel structures. After initially using a homology model based on the closed/resting state structure of the *α*1*β*3*γ*2 GABA_A_ receptor,^21^ in which we had replaced the *β*3 subunits with *β*2 subunits to guide our mutational work, we generated the final models shown in Figure 4 by directly docking RDX into the recently published structure of the *α*1*β*2*γ*2 GABA_A_ receptor in the resting state.^16^ Both the *α*1*β*3*γ*2 receptor (pdb id:6HUG) and the *α*1*β*2*γ*2 receptor (pdb id: 6X40) have been captured with picrotoxinin bound but differ somewhat in the width of the conduction pathway. The 6HUG structure is constricted to ~1.5 Å at both the desensitization gate at −2’ and the activation gate at 9’ and is therefore assumed to represent the closed/resting state. In contrast, the 6X40 structure is less tightly closed and the activation gate at the 9’ ring is partially open, suggesting that the channel was captured in a transition from the resting to the desensitized state.^16^ RosettaLigand identified low‐energy binding poses that show RDX interacting with residues at the 2’ and 6’ ring in agreement with the mutagenesis in both models, but we favored the real structure over the homology model for the final model.

Given its electrophysiological behavior as a noncompetitive antagonist and its binding to the NCA site in the pore of *α*1*β*3*γ*2 receptor, RDX is thus a “classic” GABA_A_ receptor blocker that even structurally resembles other “caged” convulsants like picrotoxinin or the rodenticide TETS (tetramethylenedisulfotetramine). So why would military personnel voluntarily consume RDX? There is, of course, the thrill of doing something as daring as “eating explosive” in front of a peer group, which based on case reports between 1969 and 2019, is particularly appealing to young males since no intentional ingestions have ever been reported in females.^9^ The practice of field troops in the Vietnam War of consuming RDX to simulate or enhance the effects of ethanol^1^ could have been based on interactions between RDX and ethanol. We here found that RDX in a concentration range of between 500 nmol/L and 2 *μ*mol/L potentiates GABA_A_ receptor activity, and it is therefore perceivable that low RDX concentrations enhance the relaxing and sedating effects of ethanol. Soldiers who had consumed RDX are described as having a reduced attention span, a reduced ability to perform simple arithmetic and being disorientated,^1^ at 24 h after exposure when they had stopped seizing and when RDX brain and plasma levels were presumably again in the range where RDX potentiates GABA_A_ receptors. At higher RDX concentrations, where RDX is a GABA_A_ receptor antagonist, there might be a small window where GABA inhibition enhances neural excitability and balances with the intoxicating, disinhibitory, and depressant effects of ethanol, an interaction which has been suggested for *α*‐thujone and ethanol in absinthe.^25^ The terpenoid *α*‐thujone, which is found in wormwood oil and in sage, inhibits GABA_A_ receptors with an IC_50_ of 21 *μ*mol/L and induces seizures,^26^ very similar to RDX. Whatever the reasons for voluntary RDX consumption, our findings suggest that RDX‐ induced seizures should be treated with GABA_A_ modulating drugs such as benzodiazepines, barbiturates, propofol, or with neurosteroids, such as alloprenanolone or ganaxolone. In several of the recent case reports where RDX had been consumed as “a dare”, seizures were treated with diazepam^8^ or lorazepam^7^, ^9^ or, on recurrence, terminated with propofol.^8^

## Conflict of Interest

None of the authors have any conflict of interest to disclose related to this work.

## Author Contributions

BP, INP, and HW developed the concept and designed the study. BP performed the electrophysiology and the molecular modeling. RDL performed the mutagenesis. VS determined the plasma protein binding. BP, RDL, and VS acquired and analyzed data. BP and HW prepared the Figures. BP and HW wrote the manuscript with input from all co‐authors.

## Supporting information


**Figure S1.** RDX acts as a positive allosteric modulator at low concentrations. (A) Comparison of the concentration response curve for picrotoxinin (PTX) and RDX on currents evoked by EC_90_ GABA for the *α*1*β*2*γ*2L GABA_A_ receptor. Data points are mean ± SD from 3 to 8 independent recordings. (B) Percentage current activation by 1 *μ*mol/L RDX (mean ± SD from 4 to 8 independent recordings) for the different GABA_A_ receptor combinations.
