# Supplementary material for: The seizure‐inducing plastic explosive RDX inhibits the α1β2γ2 GABAA receptor
Source: Ann Clin Transl Neurol. 2022 Mar 24;9(5):600–9. doi: 10.1002/acn3.51536 (PMC9082378; doi:10.1002/acn3.51536)

Supporting Information

The seizure-inducing plastic explosive RDX inhibits the α1β2γ2 $GABA_A$ receptor

Supplementary Figure 1. RDX acts as a positive allosteric modulator at low concentrations. (A) Comparison of the concentration response curve for picrotoxinin (PTX) and RDX on currents evoked by EC_{90} GABA for the α1β2γ2L $GABA_A$ receptor. Data points are mean ± SD from 3-8 independent recordings. (B) Percentage current activation by 1 μM RDX (mean ± SD from 4-8 independent recordings) for the different $GABA_A$ receptor combinations.

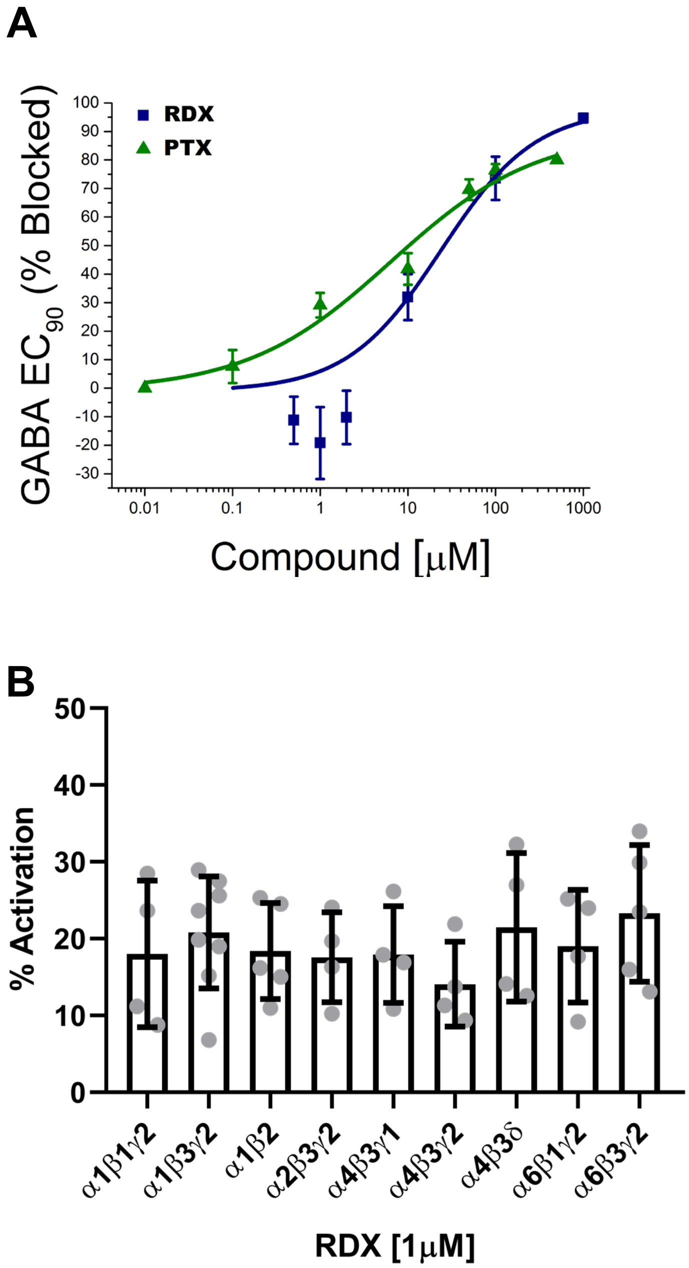

Supplement: Supplementary file 1 — Figure S1. RDX acts as a positive allosteric modulator at low concentrations. (A) Comparison of the concentration response curve for picrotoxinin (PTX) and RDX on currents evoked by EC90 GABA for the α1β2γ2L GABAA receptor. Data points are mean ± SD from 3 to 8 independent recordings. (B) Percentage current activation by 1 μmol/L RDX (mean ± SD from 4 to 8 independent recordings) for the different GABAA receptor combinations. [file ACN3-9-600-s001.pdf]
